# Socioeconomic and environmental determinants of under-five mortality in Gamo Gofa Zone, Southern Ethiopia: a matched case control study

**DOI:** 10.1186/s12914-018-0153-7

**Published:** 2018-02-27

**Authors:** Girma Temam Shifa, Ahmed Ali Ahmed, Alemayehu Worku Yalew

**Affiliations:** School of Public Health, Addis Ababa University, Addis Ababa, Ethiopia

**Keywords:** Under-five mortality, Infant mortality, Childhood mortality, Determinants of under-five mortality, Gamo Gofa, Ethiopia

## Abstract

**Background:**

Despite global declaration of the right to life as a fundamental human right and substantial progress in reducing childhood mortality, unacceptably high number of children still die before their fifth birthday every day. Different factors have been studied and implicated for under-five mortality with mixed results. Mortality studies in the current study sites were lacking. Therefore, this study examined environmental and socioeconomic determinants of under-five mortality.

**Methods:**

The study applied a matched case control study design on 381 cases of children who died before their fifth birthday and 762 controls born within 1 month in the same locality as the cases. We conducted weighted conditional logistic regression to assess the association between selected factors and mortality status.

**Result:**

The odds of death was found to be significantly lower among children of mothers whose educational status was grade nine or above (Adjusted odds ratio (AOR) of 0.34(0.16–0.72)). The odds of death was significantly higher among children whose mothers’ marital status were separated/divorced or widowed (AOR of 3.60(1.23–10.47)) and whose fathers were daily laborers (AOR of 2.34(1.29–4.23)). Presence of separate kitchen in the household for cooking was a proximate factor which was significantly associated with under-five mortality with AOR of 1.77(1.16–2.70).

**Conclusion:**

Socioeconomic factors like maternal education, husband occupation and marital status of the mother were shown to be significantly associated with under-five mortality. Hence, in order to enhance reduction in childhood mortality, investing on maternal education targeting those at risk groups is recommended.

## Background

The right to life is declared to be a fundamental human right [1]. It is the obligation of nations and governments to foster conditions essential for life and survival [1] to curb preventable deaths. Even though substantial progress has been made since the 1990s in reducing childhood mortality globally, every day, high number of children still die before celebrating their fifth birthday [2]. In Ethiopia, although child mortality substantially decreased since the 1990s, child mortality rate in the country remains to be among the highest in the world. As a result, about one in every 21 Ethiopian children dies before his/her first birthday, and one in every 15 children dies before their fifth birthday [3]. Further discussion of trends and situation of under-five mortality in Ethiopia is presented elsewhere [4].

Different factors have been studied and implicated for the continued high rate of under-five mortality in the world in general and in developing countries in particular, with mixed results. For example, the wealth status of a household is generally expected to be inversely associated with childhood mortality. This has as well been supported by findings of different studies [5–7]. However, a number of studies in developing countries showed a non-significant effect of socio-economic status of a given individual household on under-five mortality [8–14].

Access to safe drinking water and improved sanitation facilities are household characteristics which are generally considered as part of strategies to reduce infant and child mortality [15, 16]. In congruence with this notion, positive association between presence of a latrine in the household and childhood mortality was reported by different studies [17, 18]. On the other hand, findings contradicting with this fact were reported by others [11, 12, 14].

Similarly, the total number of household members (household size) is assumed to influence childhood mortality; however, the expected effect of this variable has not been uniform or clear. A larger number of household members could imply higher fertility levels and a fiercer competition for resources there by increasing the risk of childhood mortality. But, it may also imply that a larger number of potential caregivers residing in the household, thereby decreasing the risk of mortality as a study by Uddin et al. has shown [19].

It has been frequently argued that maternal education, assumed to be a vital indicator of maternal status in the household, is an important factor explaining risk of infant and child mortality. For example, maternal education was shown to positively affect child survival by many studies [18, 20–23] with a notable effect among older children than neonates [22]. Several hypotheses have been suggested to explain this association. It is postulated that maternal education inculcates modern health knowledge, beliefs and practices; improves the effectiveness of health behavior (feeding practices, child care etc.); and changes a mother’s role within the family, enabling her to take the necessary measures to prompt child health, including effective use of modern health services [24].

Besides, mothers are more likely to use scarce resources for the benefit of their children if they are free to do so [25]. Mothers with greater autonomy may also benefit in other ways that indirectly affect their children. For example, they make greater use of antenatal care (ANC), institutional delivery and postnatal care services [26] and contraceptives [27]. However, the effect of maternal autonomy on health service utilization was shown to vary on socio-economic status of the region where the mother was living [26]. Besides, the findings were not universal that, unclear correlation between women’s empowerment (participation in decision-making processes) in the household and childhood mortality was reported by another study [22].

Similarly, many hypotheses could be stipulated with regard to the association between maternal working status and survival status of her child. A working mother may have a good income to pay for food and other health interventions which may positively affect survival of the child. On the other hand, working mothers may lack time to care for their children’s health; and this may negatively affect survival of the children. A study by Kishore and Parasuraman [28] showed that there was negative relationship between maternal work status and infant and child survival, especially for male children.

Besides such mixed reports on determinants of childhood mortality, determinant factors of childhood mortality may vary through time as a result of changes in life style of nations, partly due to globalization. This may demand continuous investigation of different categories of factors identified as determinants in the past, by including emerging factors or factors which had contradicting results. Therefore, this study investigated environmental and socioeconomic determinants of under-five mortality in Gamo Gofa Zone, southern Ethiopia, to identify areas to be focused on in order to sustain the reduction of childhood mortality and improve survival.

## The conceptual framework

**Fig. 1:**
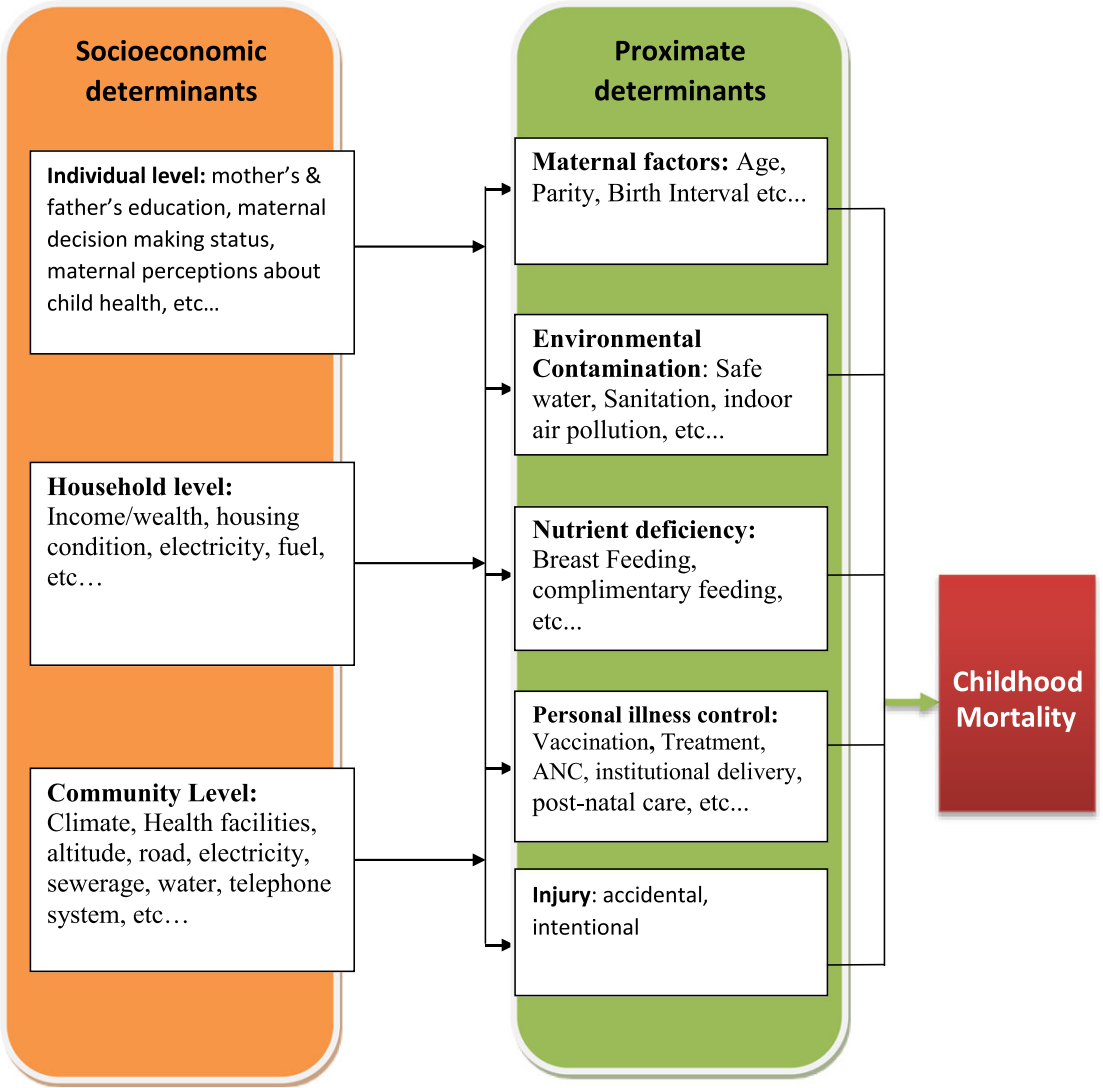
Conceptual framework, showing the operation of proximate and socioeconomic determinants of under-five mortality (adapted from Henry Mosley and Lincoln Chen’s analytic framework for the study of determinants of childhood mortality)

The analysis of the association between socioeconomic and environmental related variables and under-five mortality in this study was determined using Henry Mosley and Lincoln Chen’s analytic framework for the study of determinants of childhood mortality [29]. According to this framework, socio-economic variables operate through a set of proximate determinants that directly influence the risk of disease and outcome of disease processes. The framework identifies three categories of distal factors (individual, household and community level). The framework also defines the following five categories of proximate determinants of childhood mortality: 1) Maternal factors, 2) Environmental Contamination, 3) Nutrient deficiency, 4) Personal illness control and 5) Injury. In this study, community level factors were controlled at design level by matching the cases and controls by their place of residence. This paper focused on the distal (socioeconomic) factors and environmental contamination related factors, as the other proximate factors were the focus of another paper of the authors. The adapted conceptual framework for the overall determinants of childhood mortality is summarized in Fig. 1.

## Method

### Study area

The study area, Gamo Gofa Zone of southern Ethiopia, has 15 districts (locally known as woredas) and two town administrations. Arba Minch Town is the capital of Gamo Gofa Zone and is located 502 km south of Addis Ababa (the capital city of Ethiopia). The zone is known for its abundant banana, apple and fish production that may impact child nutrition thereby child survival. Three hospitals and 68 health centers were providing health services in the zone during the study period. During the survey, the total population of Gamo Gofa was projected to be 1,901,953 (with 285,043 urban (15%) and 1,616,910 rural (85%) residents) [30]. The study was conducted in one rural district (Arba Minch Zuria District) and one town administration (Arba Minch Town) of the zone. Arba Minch Zuria District was selected as it is the study site of the Arba Minch Demographic Surveillance System (DSS), which is relatively a new site in the country. Additionally, this district has three climatic/ geographic zones (Dega (high land), Woina dega (mid land) and Kolla(low land)); which enables representation of population from different agro-ecological zones. Arba Minch Zuria has 29 kebeles (lowest administrative units in Ethiopia) with a total population of 185,302 (with 92,680 males and 92,622 female) [30]. Arba Minch Town was included to represent the urban population of the zone. The Town is divided into 11 urban kebeles with a total population of 135,452 (with 68,132 males and 67,320 female) [30]. Further details of the study area are presented elsewhere [4].

### Study design and period

The study applied a matched case control design where under-five children who died before their 5^th^ birthdate between March 01, 2011 and September 30, 2014 were taken as cases and two controls of alive children who were born within 1 month of age and in the same locality for each case, as controls.

### Sample size determination

The sample size was calculated using the statcalc command of Epi info 7 statistical software package. The prevalence of exposures (selected determinants) among controls and odds ratios (OR) were estimated from previous studies [31–34]. Then, the sample size required for detecting an estimated odds ratio of at least 1.75, with a two-sided probability of type I error of 5% and a power of 80% was determined for all main variables with control to case ratio of two. Then, the maximum sample size was taken. Accordingly, the sample size corresponding to wealth index (prevalence of low wealth index among controls estimated to be 73.9% [32]) was taken. Hence, a minimum of 241 cases and 482 controls were required. By applying a design effect of 1.5 and adding 5% to compensate for expected non-response, a total of 380 cases and 760 controls were required.

### Sampling technique

Arba Minch Town and the Arba Minch Zuria District were selected purposively out of the 15 districts and two Town administrations of the Zone. Then, census of all the 11 kebeles of Arba Minch Town and randomly selected 11 non-DSS kebeles of Arba Minch Zuria District was conducted. Besides, data from nine kebeles of Arba Minch DSS were included (initially those kebeles were selected randomly out of 29 kebeles of the Arba Minch Zuria District). The Arba Minch DSS has been tracking all births and deaths since its establishment in 2009. Accordingly, 31 kebeles from the two districts were included in this study (*details of this part is presented elsewhere* [4]). From the census and the Arba Minch DSS, 383 cases that fulfilled the inclusion criteria were identified. All of them with their corresponding matched and randomly selected 766 controls were approached.

### Data collection

A pre-tested closed-ended structured Amharic questionnaire was utilized for data collection. The questionnaire was developed in English, based on literature and previous pertinent experiences. It was translated into Amharic, then back translated to English, to ensure its consistency. Finally, the Amharic Version was used for data collection. Two data collectors (at least certificate holders after completing grade 10) per kebele were recruited and trained on the procedure. Four supervisors who had master’s degree supervised the data collection. The principal investigator strictly followed the data collection process.

### Measurements and operational definitions

***Dependent variable:*** Mortality status (dead or alive) of the index child.

***Independent variables:*** Socioeconomic characteristics, such as education, ethnicity, religion, occupation, marital status, wealth index, maternal decision power in the household and environmental contamination related factors, such as source of drinking water and light, presence of a separate kitchen and window, whether animals were living with people in the same house or not, presence and types of latrines etc.

***Wealth index:*** was computed using principal component analysis (PCA) of reported ownership of household assets such as radio, television, bicycle, livestock, etc. and proxy indicators of living standard variables, like number of rooms the living house had, the roof of the house, whether the house was private or rental etc. (for urban and rural separately). Then, using quintiles the wealth status was categorized into three groups (poor, average and rich). The 1^st^ and 2^nd^ quintiles were grouped as poor and the top 4^th^ and 5^th^ quintiles were classified as rich.

***Maternal power:*** was also computed using PCA of eight variables that, measure decision making power of the mother in the household (decision on maternal and husband income, on purchase and on visiting relatives or health facility (1 was assigned, if the response favored maternal power (eg. 1 if mother decided by herself or jointly with her partner) other wise 0), perception on wife beating (0 if she justified to at least for one of the options listed), if ever beaten by husband (0 if ever beaten), if husband assisted in household chores (0 if husband didn’t assist)). Then, using quintiles maternal decision making status was categorized in to three (poor, average and good). The 1^st^ and 2^nd^ quintiles were grouped as poor and the top 4^th^ and 5^th^ quintiles were classified as good.

**Infant mortality**: is the probability of dying between birth and the first birth day.

**Under five mortality:** is the probability of dying before the fifth birthday.

### Data processing and management

The data were edited, coded, entered into computer and cleaned using Epi Info Version 3.5.1. We analyzed the data using SPSS version 16 and/or STATA version 11 as appropriate. The daily collected data/questionnaires were transferred to the Arba Minch University on daily basis. Supervisors and principal investigator checked completeness and appropriateness of all raw data before acceptance as completed and kept in locked cabinet. Two experienced and trained data encoders entered the data into Epi info. The principal investigator strictly followed data entry and checked the data on daily basis. Ten percent of the questionnaires were double entered to check discrepancies.

### Data analysis

To determine the effect of distal (socio-economic) and proximate (environmental contamination) factors on mortality of the child, we conducted weighted conditional logistic regression. Sampling weight taken as the reciprocal of probabilities of selection was calculated as follows. The probability of selection for urban was = 1/2*1*306/5745 = 0.027 (as one out of two urban districts and all the kebeles in selected district were included; and 306 out of 5745 children identified by the census were included). The corresponding sampling weight for urban was 37.6. For that of rural =1/15*20/29*837/14416 = 0.0027(as one out of 15 rural districts and 20 out of 29 kebeles of the district were included; and 837 out of 14,416 children identified were included in the study). The corresponding sampling weight for rural was 374.6.

We fitted two consecutive models based on the Mosley & Chen’s analytical framework for the study of determinants of childhood mortality, first for under-five children, and then for infants [29]. According to Mosley & Chen’s analytical framework, distal factors like socioeconomic factors operate through proximate factors, such as environmental contamination factors, to affect childhood mortality. Accordingly, we first fitted model for distal factors for under-five children and infants. Then, we controlled those distal factors which were significant at *p*-value of 0.1 for models of proximate variables (environmental contamination factors). Sex of the child was also controlled in all models.

Model diagnostic was conducted following each model using different STATA commands. Presence of specification error in the link term (logistic) or predictors was assessed with linktest command. Besides, the goodness of fit of the models was assessed by log likelihood chi-square test and Akaike Information Criterion (AIC) using estat ic command. Presence of interaction/effect modification was also assessed for suspected variables in both models. Multicollinearity among predictors was assessed for each model and tolerance value <=0.1 or variance inflation factor (VIF) of > = 10 was taken as an indicator of presence of multicollinearity.

## Result

### Socioeconomic-demographic characteristics of the respondents

All the 383 cases identified from the census and the Arba Minch DSS and their corresponding 766 controls residing in the 11 urban and 20 rural study kebeles were approached for this study, and data were collected from 381 cases and 762 controls. Data from two cases and their corresponding controls were not availed since the respective cases migrated out from their initial locations.

As displayed in Table 1 below, 210(55.1%) of cases and 382(50.1%) of controls were males. Majority of the mothers of cases 332(87.1%) and controls 688(90.3%) were aged between 18 and 35 years. Most of the mothers of cases 251(65.9%) and controls 517(67.8%) were Protestant Christians. With regard to maternal education, more than half, 210(55.1%) of the cases and less than half 362(47.5%) of the controls had no formal education.

**Table 1:** Socioeconomic-demographic characteristics of the participants, Gamo Gofa Zone, 2014

Characteristics	Survival status of the child		*P*-value*	Total *n*(%)
	Dead *n*(%)	Alive *n*(%)		
Sex of the child				
Male	210(55.1)	382(50.1)	0.112	592(51.8)
Female	171(44.9)	380(49.9)		551(48.2)
Maternal age				
< 18 year	7(1.8)	12(1.6)	0.257	19(1.7)
18-35 year	332(87.1)	688(90.3)		1020(89.2)
> 35 year	42(11.0)	62(8.1)		104(9.1)
Mother Religion				
Protestant	251(65.9)	517(67.8)	0.797	768(67.2)
Orthodox	115(30.2)	216(28.3)		331(29.0)
Others	15(3.9)	29(3.8)		44(3.8)
Mother education				
No formal education	210(55.1)	362(47.5)	< 0.001	572(50.0)
Grade1–6	106(27.8)	188(24.7)		294(25.7)
Grade7–8	25(6.6)	80(10.5)		105(9.2)
Grade 9 and above	40(10.5)	132(17.3)		172(15.0)
Mother ethnicity				
Gamo	286(75.1)	571(74.9)	0.990	857(75.0)
Gofa	8(2.1)	20(2.6)		28(2.4)
Wolayta	26(6.8)	52(6.8)		78(6.8)
Zeyse	37(9.7)	78(10.2)		115(10.1)
Amhara	6(1.6)	19(2.5)		25(2.2)
Others	18(4.7)	22(2.9)		40(3.5)
Mother Occupation				
Farmer	22(5.8)	35(4.6)	0.384	57(5.0)
House wife	264(69.3)	539(70.7)		803(70.3)
Gov’t employee	14(3.7)	36(4.7)		50(4.4)
Merchant	36(9.4)	84(11.0)		120(10.5)
Others	45(11.8)	68(8.9)		113(9.9)
Marital status				
Married	358(94.0)	731(95.9)	0.102	1089(95.3)
Single	11(2.9)	21(2.8)		32(2.8)
Others(separated/divorced or widowed)	12(3.1)	10(1.3)		22(1.9)
Husband occupation				
Farmer	190(50.1)	433(56.8)	< 0.001	623(54.5)
Gov’t or NGO employee	34(8.9)	96(12.6)		130(11.4)
Merchant or self-employee	33(8.7)	73(9.6)		106(9.3)
Daily laborer	66(17.3)	61(8.0)		127(11.1)
Student	6(1.6)	15(2.0)		21(1.8)
Others	29(7.6)	53(7.0)		82(7.2)
No husband	23(6.0)	31(4.1)		54(4.7)
Maternal power				
Poor	160 (42.0)	290(38.1)	0.380	450(39.4)
Average	19(5.0)	35(4.6)		54(4.7)
Good	202(53.0)	437(57.3)		639(55.9)
Wealth index of the household				
Poor	159(41.7)	298(39.1)	< 0.001	457(40.0)
Average	98(25.7)	136(17.8)		234(20.5)
Rich	124(32.5)	328(43.0)		452(39.5)

*The p-values are determined using Pearson chi-square

Ethnically, majority of the mothers of cases 286(75.1%) and controls 571(74.9%) were from Gamo ethnic group. Most of the mothers of cases 264(69.3%) and controls 539(70.7%) were housewives. Majority of the mothers of cases 358(94.0%) and controls 731(95.9%) were married. Nearly half of the husband of cases 190(50.1%) and controls 433(56.8%) were farmers while the rest comprised of daily laborers, merchants, government employees etc. With regard to maternal decision power, 202(53.0%) of the cases and 437(57.3%) of the controls had good decision power. In terms of wealth index, only 124(32.5%) of the cases were classified as rich, whereas, 328(43.0%) of the controls were classified as rich (Table 1).

### Association of socioeconomic characteristics (distal factors) with under-five mortality

As it is shown in Table 2 (model 1), the adjusted weighted conditional logistic regression revealed that among distal factors maternal education, husband occupation and marital status of the mother were factors significantly associated with under-five and/or infant mortality.

**Table 2:** Association of Socioeconomic-demographic characteristics (distal factors) with child mortality, Gamo Gofa Zone, 2014

Characteristics	Categories	Under-five^a^			Infants^a^		
		AOR	[95% Conf. Interval		AOR	[95% Conf. Interval	
Sex of the child	Male	Ref					
	Female	0.76	0.57	1.02	0.75	0.52	1.08
Mother’s religion	Protestant	Ref					
	Orthodox	1.27	0.86	1.87	1.10	0.67	1.79
	Others	1.19	0.53	2.67	1.40	0.41	4.71
Mother’s Ethnicity	Gamo	Ref					
	Zeyse	1.09	0.38	3.17	0.62	0.14	2.82
	Wolayta	1.08	0.48	2.40	0.66	0.25	1.76
	Others	1.66	0.87	3.18	1.37	0.63	2.98
Mother’s education	No formal education	Ref					
	Grade1–6	0.95	0.63	1.43	0.97	0.57	1.65
	Grade7–8	0.67	0.35	1.31	0.69	0.31	1.56
	Grade 9 and above	**0.34**	**0.16**	**0.72**	**0.37**	**0.15**	**0.91**
Mother’s Occupation	Farmer	Ref					
	House Wife	0.70	0.32	1.52	0.59	0.25	1.38
	Gov’t employee	0.57	0.12	2.76	0.80	0.14	4.61
	Merchant	0.50	0.20	1.28	0.43	0.15	1.28
	Others	1.31	0.44	3.90	1.10	0.33	3.66
Marital status	Married	Ref					
	Single	1.22	0.42	3.54	0.49	0.11	2.30
	Others(separated/divorced or widowed)	**3.60**	**1.23**	**10.47**	**6.68**	**1.81**	**24.71**
Husband occupation	Farmer	Ref					
	Gov’t employee	0.70	0.31	1.55	0.78	0.30	2.03
	Merchant	1.84	0.92	3.66	1.43	0.66	3.08
	Daily laborer	**2.34**	**1.29**	**4.23**	1.79	0.86	3.74
	Student	1.27	0.42	3.87	1.47	0.32	6.77
	Others	1.67	0.86	3.24	1.54	0.69	3.44
Wealth index of Household	Rich	Ref					
	Average	1.49	0.85	2.62	1.51	0.74	3.07
	Poor	1.52	0.93	2.48	1.75	0.97	3.16
Number of individuals living in the house		0.94	0.87	1.03	0.93	0.83	1.03
Maternal power	Poor	Ref					
	Average	1.48	0.69	3.19	1.88	0.80	4.44
	Good	0.91	0.65	1.28	0.89	0.57	1.38

^a^Adjusted for all variables in the table and all bold values are statistically significant at 0.05

The odds of death among under-five children of mothers whose educational status was grade nine or above was 66% (adjusted odds ratio (AOR) of 0.34(0.16–0.72)) lower than among children of mothers who lacked formal education. Similarly, educational status of the mother was shown to be inversely associated with infant mortality (AOR of 0.37(0.15–0.91)) (Table 2).

The odds of death among under-five children whose mothers’ marital status were in the others category (separated/divorced or widowed) was about 4 times (AOR of 3.60(1.23–10.47)) higher than among those whose mothers were married. Similarly, infants whose mothers’ marital status were in other category (separated/divorced or widowed) had about 7 times (AOR of 6.68(1.81–24.71)) higher rate of odds of death than those whose mothers were married (Table 2).

Under-five children whose fathers were daily laborers had more than two times (AOR of 2.34(1.29–4.23)) higher rate of odds of death than those whose fathers were farmers. Though it was not significant, infants whose fathers were daily laborers had about 1.8 times (AOR of 1.79(0.86–3.74)) higher rate of odds of death than those whose fathers were farmers (Table 2).

Though the association between wealth index of the household and under-five mortality was in the expected direction (children of poor families had a higher rate of odds of death), it was not statistically significant with AOR of 1.49(0.85–2.62) for average and AOR of 1.52(0.93–2.48) for poor when compared with rich categories. Index of maternal power in the household was also not significantly associated with under-five mortality with AOR of 1.48(0.69–3.19) for average and AOR of 0.91(0.65–1.28) for good when compared with the poor categories. Other distal factors which did not show statistically significant association with under-five and infant mortality were family size, mother’s occupation, religion and ethnicity of the mother (Table 2).

### Association of environmental contamination (proximate) factors with under-five mortality

Among factors classified as environmental contamination, presence of separate kitchen was significantly associated with both under-five and infant mortality. The odds of death among under-five children from a household which lacked separate kitchen for cooking was about 1.8 times (AOR of 1.77(1.16–2.70)) higher than among those with separate kitchen. Similarly, infants from households which lacked separate kitchen had about 1.9 times (AOR of 1.94(1.13–3.33)) higher rate of odds of death than those with separate kitchen. The odds of death among infants whose household’s source of light was electricity was less (with AOR of 0.47(0.23–0.99)) than among those whose source was other than electricity (Table 3).

**Table 3:** Association between environmental contamination factors & under-five mortality, Gamo Gofa Zone, 2014

Characteristics	Under-five^a^			Infants^b^		
	AOR	[95% Conf. Interval]		AOR	[95% Conf. Interval]	
The house has window						
Yes	RF					
No	0.74	0.45	1.23	0.75	0.40	1.40
Did animals live with people						
Yes	Ref					
No	1.17	0.72	1.92	1.07	0.60	1.90
Had separate kitchen						
Yes	Ref					
No	**1.77**	**1.16**	**2.70**	**1.94**	**1.13**	**3.33**
Type of latrine the family had						
No latrine	Ref					
Pit latrine	1.03	0.60	1.76	1.04	0.51	2.11
VIP/Flush	1.37	0.50	3.73	1.04	0.30	3.65
Main source of water						
Tap	Ref					
Protected well/spring	0.92	0.51	1.66	1.48	0.69	3.20
Unprotected well/spring/river/pond	1.02	0.61	1.72	1.26	0.63	2.52
Source of light of the household						
Other than electricity	Ref					
Electricity	0.87	0.48	1.57	**0.47**	**0.23**	**0.99**

^a^Besides variables in the table adjusted for sex of the child, mother’s education, wealth index, husband occupation and marital status of the mother

^b^Besides the variables in the table adjusted for sex of the child, mother’s education, wealth index and marital status of the mother and all bold values are statistically significant at 0.05

Surprisingly, presence or type of latrine was not significantly associated with under-five mortality with AOR of 1.03(0.60–1.76) for pit latrine and AOR of 1.37(0.50–3.73) for ventilated and improved pit (VIP)/ flush toilet when compared to no latrine. Similarly, source of water was not significantly associated with under-five mortality with AOR of 0.92(0.51–1.66) for protected well/spring and AOR of 1.02(0.61–1.72) for unprotected well/spring/river/pond when compared with tap water. Environmental contamination related factors, like presence of window and sharing the house with animals were also not significantly associated with both under-five and infant mortality (Table 3).

## Discussion

This study tried to identify socioeconomic and environmental determinants of childhood mortalities by aggregating predicting variables into relevant levels (distal or proximal factors) in order to determine unbiased effects of identified determinants. To achieve this, among others, the study employed efforts like matching of cases and controls at design stage and employment of weighted conditional logistic regression at analysis stage.

However, as information was collected retrospectively systematic errors such as recall and social desirability biases may affect some of the findings. Besides, in controlling confounding factors, especially in the second model (for environmental contamination related factors), the study may not be exhaustive in addressing other confounding factors. However, it is possible to assume that the confounding effect of proximate variables could be at least partially controlled by controlling distal factors, which are assumed to be operating through those proximate factors.

Among distal factors, maternal education was shown to be important determining factor of under-five and infant mortality. Both infants and under-five children of mothers who were at least grade nine had less odds of death than those without any formal education. This finding is in line with many other studies conducted in different parts of the world [18, 20–23, 35]. This may be because of the effect of maternal education on modern health knowledge, beliefs and practices of the mother, that in turn could foster effectiveness of health behavior (feeding practices and child care); and changes the mother’s role in the family by enabling her to take necessary measures, including better use of modern health services such as immunization, ANC, delivery, postnatal care and contraceptives [24, 26, 27, 36].

Another important distal factor identified as a predicting factor of under-five mortality was husband’s occupation. Children whose fathers were daily laborers had higher rate of odds of death than those whose fathers were farmers. This may be due to the fact that occupation of a father is an indicator of socioeconomic status of the household and stability of the family. It is logical to expect being a farmer is economically better and stable than being daily laborer; provided that majority of the current study population were from rural kebeles.

Both infants and under-five children whose mothers were separated/ divorced or widowed were more likely to have higher rate of odds of death than those whose mothers were married. This was in line with other studies done in Africa, that children of married mothers were more likely to survive than unmarried ones [8, 17, 37]. This may be for obvious reason that, these mothers are more likely to be economically deprived of than the ones with husbands, because of lack of support from the husband, who are the one responsible for income of the household. Besides, married mothers are likely to get support from their partners for utilization of health services during antenatal through postnatal care. The insignificant effect of being single on under-five mortality observed in this study may be because of the fact that single mothers are more likely to live with their parents and get the support to care for their children.

In this study, unexpectedly, wealth index of the household was not significantly associated with under-five mortality in contrast to other studies [5–7]. However, the current finding is in line with studies in developing countries which showed non-significant effect of socioeconomic status of an individual household on under-five mortality [8–14]. This may be because of the way socioeconomic status is defined and determined by use of principal component analysis of possession of household assets. There may be under reporting of household assets by the study participants as this is usually perceived to be linked with taxation and eligibility for social supports by the government and non-government organization in the community.

Maternal decision power as measured in the current study was not statistically significantly associated with under-five mortality. This was in contrast to previous arguments of positive effect of maternal status on childhood mortality through its effect on her decision on child health [25, 38–40]. However, unclear correlation between women’s empowerment (participation in decision-making processes) in the household and childhood mortality was reported by another study [22]. This may imply that, mere decision power of the mother may not have effect on childhood mortality unless it is supported by awareness about her and her child’s health. However, the difference may also be because of the difference in defining maternal power. Educational status of the mother is a crucial element of her status in the household, but it was treated independently in the current study.

In developing countries, traditional use of household energy like cooking and heating with biomass fuels/coal is posing a serious threat to health by producing a variety of health damaging pollutants [41] and is shown to be a risk factor for many causes of childhood mortalities [42, 43]. Among factors classified as environmental contamination; presence of separate kitchen for cooking was significantly associated with under-five mortality in the current study. Similar effect was observed among infants. The odds ratio identified in the current study might be an underestimate, as the child is expected to be at the back of his/her mother during cooking even though she is cooking in a separate kitchen. Almost all (99.7%) of households of the current study were using wood, animal dung or charcoal as source of fuel for cooking.

Presence or type of latrine facility the household had and source of drinking water were not significantly associated with childhood mortality in this study. This finding was against others’ arguments [15, 16] and findings [17, 18] of positive association between latrine facility and childhood mortality. However, our finding is in line with other studies [11, 14, 44]. This may be because of the fact that majority of latrine types in the study area were traditional pit latrines (92% were pit latrine) which are usually unclean. These type of latrines are prone to contamination by vectors like flies which may pose risk even for those children from households having improved latrine as these children are more likely to share at least the same playgrounds in a rural community. The other possible explanation may be, better access of curative health services at community level for illnesses such as diarrhea, which may arise from lack of latrine facilities or safe drinking water, as all the kebeles under the current study had a health post staffed with at least one health extension worker. However, the effect of these factors on intermediate cause of child mortality, such as diarrheal disease should not be ignored as their effect on non-death outcomes of the child’s health (nutritional status, growth, mental development etc.) is of paramount importance [45, 46].

## Conclusions

This study tried to identify socioeconomic and environmental contamination related factors of childhood mortality in the study area or in areas with similar setup by controlling potential confounding factors at design stage and during analysis. In this study, distal factors like maternal education, husband’s occupation and marital status of the mother were shown to significantly affect under-five mortality. So, in order to maintain the reduction in childhood mortality, investing on maternal education by targeting those at risk groups is required.

Among factors classified as environmental contamination; presence of separate kitchen for cooking was significantly associated with low under-five mortality. Besides, using electricity as a source of light was shown to significantly reduce infant mortality. So, in line with production and distribution of electricity to reach all households in rural areas, promotion of having a separate kitchen for cooking is important, as majority of the households are using traditional household energy sources like biomass fuels for cooking.

Even though presence or type of latrine facility the household had and source of drinking water were not significantly associated with childhood mortality in this study, effect of these factors on intermediate causes of child morbidity and mortality such as diarrheal diseases should not be ignored. Their effect on other non-death outcomes of the child’s health like nutritional status, growth and mental development is of paramount importance.

## Abbreviations

ANC: Antenatal care
AOR: Adjusted odds ratio
DSS: Demographic surveillance site
PCA: Principal component analysis
VIP: Ventilated and improved pit
